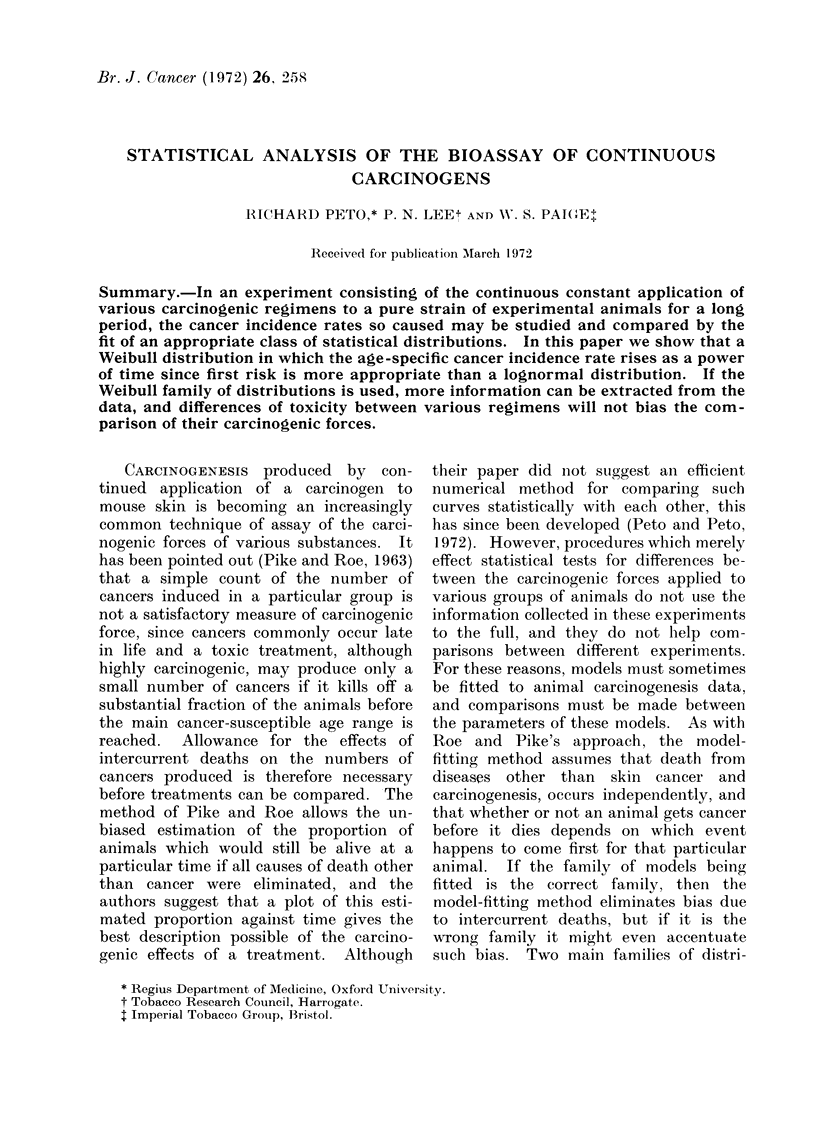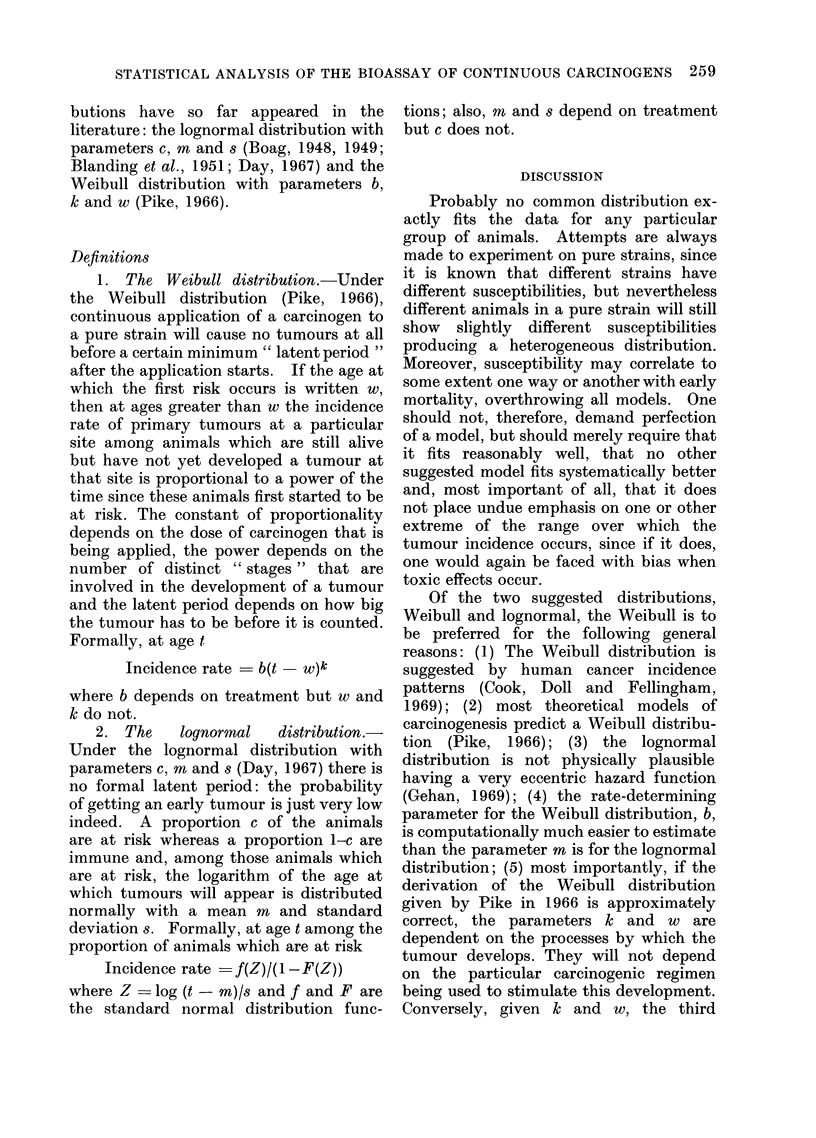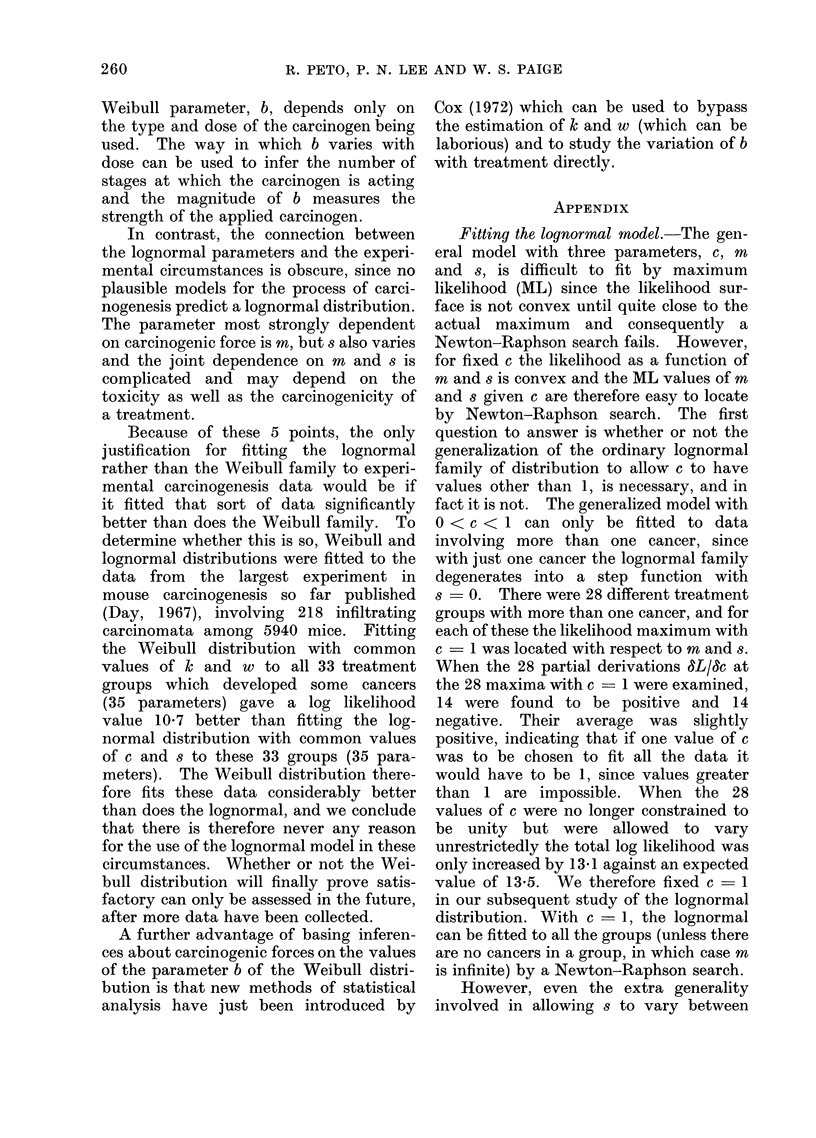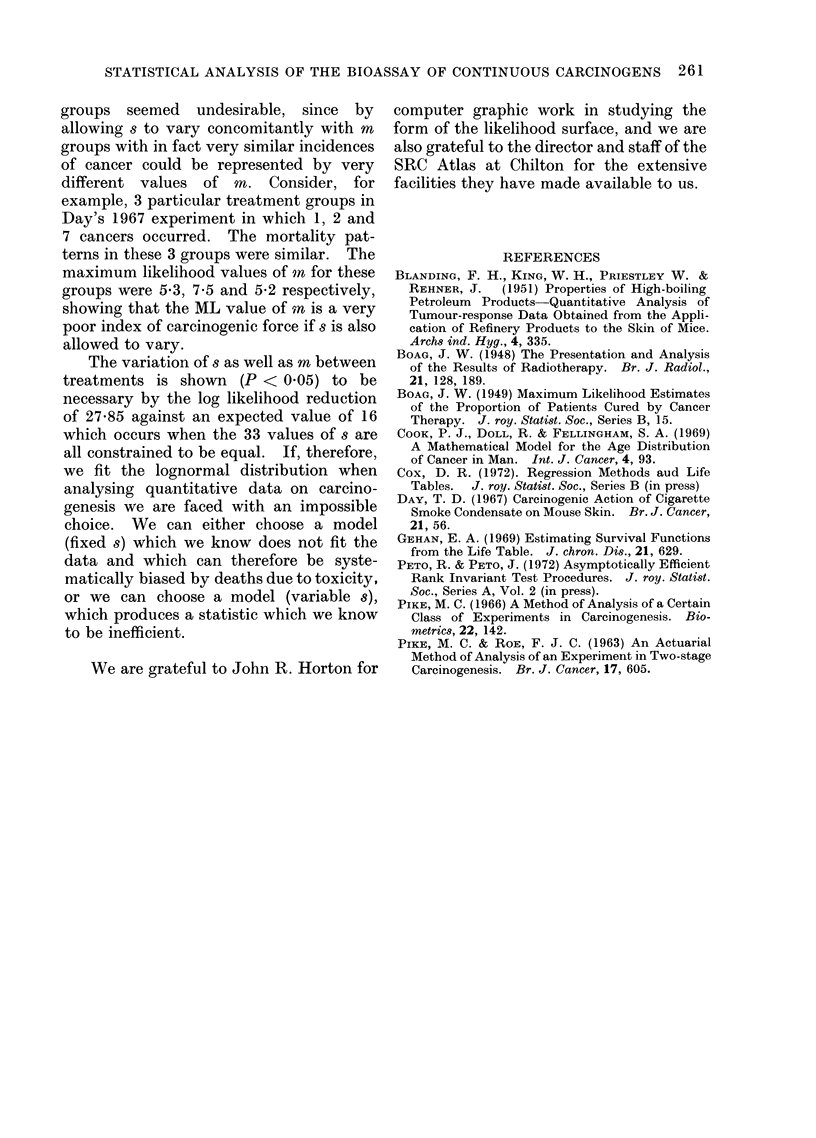# Statistical Analysis of the Bio-assay of Continuous Carcinogens

**DOI:** 10.1038/bjc.1972.34

**Published:** 1972-08

**Authors:** Richard Peto, P. N. Lee, W. S. Paige

## Abstract

In an experiment consisting of the continuous constant application of various carcinogenic regimens to a pure strain of experimental animals for a long period, the cancer incidence rates so caused may be studied and compared by the fit of an appropriate class of statistical distributions. In this paper we show that a Weibull distribution in which the age-specific cancer incidence rate rises as a power of time since first risk is more appropriate than a lognormal distribution. If the Weibull family of distributions is used, more information can be extracted from the data, and differences of toxicity between various regimens will not bias the comparison of their carcinogenic forces.


*Br. J. Cancer* (1972) **26**, 258

### STATISTICAL ANALYSIS OF THE BIOASSAY OF CONTINUOUS CARCINOGENS

RICHARD PETO,* P. N. LEE† AND W. S. PAIGE‡

Received for publication March 1972

**Summary.—In an experiment consisting of the continuous constant application of various carcinogenic regimens to a pure strain of experimental animals for a long period, the cancer incidence rates so caused may be studied and compared by the fit of an appropriate class of statistical distributions. In this paper we show that a Weibull distribution in which the age-specific cancer incidence rate rises as a power of time since first risk is more appropriate than a lognormal distribution. If the Weibull family of distributions is used, more information can be extracted from the data, and differences of toxicity between various regimens will not bias the comparison of their carcinogenic forces.**

CARCINOGENESIS produced by continued application of a carcinogen to mouse skin is becoming an increasingly common technique of assay of the carcinogenic forces of various substances. It has been pointed out (Pike and Roe, 1963) that a simple count of the number of cancers induced in a particular group is not a satisfactory measure of carcinogenic force, since cancers commonly occur late in life and a toxic treatment, although highly carcinogenic, may produce only a small number of cancers if it kills off a substantial fraction of the animals before the main cancer-susceptible age range is reached. Allowance for the effects of intercurrent deaths on the numbers of cancers produced is therefore necessary before treatments can be compared. The method of Pike and Roe allows the unbiased estimation of the proportion of animals which would still be alive at a particular time if all causes of death other than cancer were eliminated, and the authors suggest that a plot of this estimated proportion against time gives the best description possible of the carcinogenic effects of a treatment. Although their paper did not suggest an efficient numerical method for comparing such curves statistically with each other, this has since been developed (Peto and Peto, 1972). However, procedures which merely effect statistical tests for differences between the carcinogenic forces applied to various groups of animals do not use the information collected in these experiments to the full, and they do not help comparisons between different experiments. For these reasons, models must sometimes be fitted to animal carcinogenesis data, and comparisons must be made between the parameters of these models. As with Roe and Pike's approach, the model-fitting method assumes that death from diseases other than skin cancer and carcinogenesis, occurs independently, and that whether or not an animal gets cancer before it dies depends on which event happens to come first for that particular animal. If the family of models being fitted is the correct family, then the model-fitting method eliminates bias due to intercurrent deaths, but if it is the wrong family it might even accentuate such bias. Two main families of distri-

* Regius Department of Medicine, Oxford University.
† Tobacco Research Council, Harrogate.
‡ Imperial Tobacco Group, Bristol.

butions have so far appeared in the literature: the lognormal distribution with parameters $c$, $m$ and $s$ (Boag, 1948, 1949; Blanding *et al.*, 1951; Day, 1967) and the Weibull distribution with parameters $b$, $k$ and $w$ (Pike, 1966).

*Definitions*

1. *The Weibull distribution.*—Under the Weibull distribution (Pike, 1966), continuous application of a carcinogen to a pure strain will cause no tumours at all before a certain minimum " latent period " after the application starts. If the age at which the first risk occurs is written $w$, then at ages greater than $w$ the incidence rate of primary tumours at a particular site among animals which are still alive but have not yet developed a tumour at that site is proportional to a power of the time since these animals first started to be at risk. The constant of proportionality depends on the dose of carcinogen that is being applied, the power depends on the number of distinct " stages " that are involved in the development of a tumour and the latent period depends on how big the tumour has to be before it is counted. Formally, at age $t$

$$\text{Incidence rate} = b(t - w)^k$$

where $b$ depends on treatment but $w$ and $k$ do not.

2. *The lognormal distribution.*—Under the lognormal distribution with parameters $c$, $m$ and $s$ (Day, 1967) there is no formal latent period: the probability of getting an early tumour is just very low indeed. A proportion $c$ of the animals are at risk whereas a proportion $1\text{–}c$ are immune and, among those animals which are at risk, the logarithm of the age at which tumours will appear is distributed normally with a mean $m$ and standard deviation $s$. Formally, at age $t$ among the proportion of animals which are at risk

$$\text{Incidence rate} = f(Z)/(1-F(Z))$$

where $Z = \log(t - m)/s$ and $f$ and $F$ are the standard normal distribution functions; also, $m$ and $s$ depend on treatment but $c$ does not.

DISCUSSION

Probably no common distribution exactly fits the data for any particular group of animals. Attempts are always made to experiment on pure strains, since it is known that different strains have different susceptibilities, but nevertheless different animals in a pure strain will still show slightly different susceptibilities producing a heterogeneous distribution. Moreover, susceptibility may correlate to some extent one way or another with early mortality, overthrowing all models. One should not, therefore, demand perfection of a model, but should merely require that it fits reasonably well, that no other suggested model fits systematically better and, most important of all, that it does not place undue emphasis on one or other extreme of the range over which the tumour incidence occurs, since if it does, one would again be faced with bias when toxic effects occur.

Of the two suggested distributions, Weibull and lognormal, the Weibull is to be preferred for the following general reasons: (1) The Weibull distribution is suggested by human cancer incidence patterns (Cook, Doll and Fellingham, 1969); (2) most theoretical models of carcinogenesis predict a Weibull distribution (Pike, 1966); (3) the lognormal distribution is not physically plausible having a very eccentric hazard function (Gehan, 1969); (4) the rate-determining parameter for the Weibull distribution, $b$, is computationally much easier to estimate than the parameter $m$ is for the lognormal distribution; (5) most importantly, if the derivation of the Weibull distribution given by Pike in 1966 is approximately correct, the parameters $k$ and $w$ are dependent on the processes by which the tumour develops. They will not depend on the particular carcinogenic regimen being used to stimulate this development. Conversely, given $k$ and $w$, the third

Weibull parameter, $b$, depends only on the type and dose of the carcinogen being used. The way in which $b$ varies with dose can be used to infer the number of stages at which the carcinogen is acting and the magnitude of $b$ measures the strength of the applied carcinogen.

In contrast, the connection between the lognormal parameters and the experimental circumstances is obscure, since no plausible models for the process of carcinogenesis predict a lognormal distribution. The parameter most strongly dependent on carcinogenic force is $m$, but $s$ also varies and the joint dependence on $m$ and $s$ is complicated and may depend on the toxicity as well as the carcinogenicity of a treatment.

Because of these 5 points, the only justification for fitting the lognormal rather than the Weibull family to experimental carcinogenesis data would be if it fitted that sort of data significantly better than does the Weibull family. To determine whether this is so, Weibull and lognormal distributions were fitted to the data from the largest experiment in mouse carcinogenesis so far published (Day, 1967), involving 218 infiltrating carcinomata among 5940 mice. Fitting the Weibull distribution with common values of $k$ and $w$ to all 33 treatment groups which developed some cancers (35 parameters) gave a log likelihood value 10·7 better than fitting the lognormal distribution with common values of $c$ and $s$ to these 33 groups (35 parameters). The Weibull distribution therefore fits these data considerably better than does the lognormal, and we conclude that there is therefore never any reason for the use of the lognormal model in these circumstances. Whether or not the Weibull distribution will finally prove satisfactory can only be assessed in the future, after more data have been collected.

A further advantage of basing inferences about carcinogenic forces on the values of the parameter $b$ of the Weibull distribution is that new methods of statistical analysis have just been introduced by Cox (1972) which can be used to bypass the estimation of $k$ and $w$ (which can be laborious) and to study the variation of $b$ with treatment directly.

### Appendix

*Fitting the lognormal model.*—The general model with three parameters, $c$, $m$ and $s$, is difficult to fit by maximum likelihood (ML) since the likelihood surface is not convex until quite close to the actual maximum and consequently a Newton–Raphson search fails. However, for fixed $c$ the likelihood as a function of $m$ and $s$ is convex and the ML values of $m$ and $s$ given $c$ are therefore easy to locate by Newton–Raphson search. The first question to answer is whether or not the generalization of the ordinary lognormal family of distribution to allow $c$ to have values other than 1, is necessary, and in fact it is not. The generalized model with $0 < c < 1$ can only be fitted to data involving more than one cancer, since with just one cancer the lognormal family degenerates into a step function with $s = 0$. There were 28 different treatment groups with more than one cancer, and for each of these the likelihood maximum with $c = 1$ was located with respect to $m$ and $s$. When the 28 partial derivations $\delta L/\delta c$ at the 28 maxima with $c = 1$ were examined, 14 were found to be positive and 14 negative. Their average was slightly positive, indicating that if one value of $c$ was to be chosen to fit all the data it would have to be 1, since values greater than 1 are impossible. When the 28 values of $c$ were no longer constrained to be unity but were allowed to vary unrestrictedly the total log likelihood was only increased by 13·1 against an expected value of 13·5. We therefore fixed $c = 1$ in our subsequent study of the lognormal distribution. With $c = 1$, the lognormal can be fitted to all the groups (unless there are no cancers in a group, in which case $m$ is infinite) by a Newton–Raphson search.

However, even the extra generality involved in allowing $s$ to vary between groups seemed undesirable, since by allowing $s$ to vary concomitantly with $m$ groups with in fact very similar incidences of cancer could be represented by very different values of $m$. Consider, for example, 3 particular treatment groups in Day's 1967 experiment in which 1, 2 and 7 cancers occurred. The mortality patterns in these 3 groups were similar. The maximum likelihood values of $m$ for these groups were 5·3, 7·5 and 5·2 respectively, showing that the ML value of $m$ is a very poor index of carcinogenic force if $s$ is also allowed to vary.

The variation of $s$ as well as $m$ between treatments is shown ($P < 0 \cdot 05$) to be necessary by the log likelihood reduction of 27·85 against an expected value of 16 which occurs when the 33 values of $s$ are all constrained to be equal. If, therefore, we fit the lognormal distribution when analysing quantitative data on carcinogenesis we are faced with an impossible choice. We can either choose a model (fixed $s$) which we know does not fit the data and which can therefore be systematically biased by deaths due to toxicity, or we can choose a model (variable $s$), which produces a statistic which we know to be inefficient.

We are grateful to John R. Horton for computer graphic work in studying the form of the likelihood surface, and we are also grateful to the director and staff of the SRC Atlas at Chilton for the extensive facilities they have made available to us.

### REFERENCES

Blanding, F. H., King, W. H., Priestley W. & Rehner, J. (1951) Properties of High-boiling Petroleum Products—Quantitative Analysis of Tumour-response Data Obtained from the Application of Refinery Products to the Skin of Mice. *Archs ind. Hyg.*, **4**, 335.

Boag, J. W. (1948) The Presentation and Analysis of the Results of Radiotherapy. *Br. J. Radiol.*, **21**, 128, 189.

Boag, J. W. (1949) Maximum Likelihood Estimates of the Proportion of Patients Cured by Cancer Therapy. *J. roy. Statist. Soc.*, Series B, 15.

Cook, P. J., Doll, R. & Fellingham, S. A. (1969) A Mathematical Model for the Age Distribution of Cancer in Man. *Int. J. Cancer*, **4**, 93.

Cox, D. R. (1972). Regression Methods aud Life Tables. *J. roy. Statist. Soc.*, Series B (in press)

Day, T. D. (1967) Carcinogenic Action of Cigarette Smoke Condensate on Mouse Skin. *Br. J. Cancer*, **21**, 56.

Gehan, E. A. (1969) Estimating Survival Functions from the Life Table. *J. chron. Dis.*, **21**, 629.

Peto, R. & Peto, J. (1972) Asymptotically Efficient Rank Invariant Test Procedures. *J. roy. Statist. Soc.*, Series A, Vol. 2 (in press).

Pike, M. C. (1966) A Method of Analysis of a Certain Class of Experiments in Carcinogenesis. *Biometrics*, **22**, 142.

Pike, M. C. & Roe, F. J. C. (1963) An Actuarial Method of Analysis of an Experiment in Two-stage Carcinogenesis. *Br. J. Cancer*, **17**, 605.